# Removing artefacts and periodically retraining improve performance of neural network-based seizure prediction models

**DOI:** 10.1038/s41598-023-30864-w

**Published:** 2023-04-11

**Authors:** Fábio Lopes, Adriana Leal, Mauro F. Pinto, António Dourado, Andreas Schulze-Bonhage, Matthias Dümpelmann, César Teixeira

**Affiliations:** 1 Center for Informatics and Systems of the University of Coimbra, Department of Informatics Engineering, University of Coimbra, Coimbra, Portugal; 2 Epilepsy Center, Department Neurosurgery, Medical Center–University of Freiburg, Faculty of Medicine, University of Freiburg, Freiburg, Germany

**Keywords:** Epilepsy, Biomedical engineering

## Abstract

The development of seizure prediction models is often based on long-term scalp electroencephalograms (EEGs) since they capture brain electrical activity, are non-invasive, and come at a relatively low-cost. However, they suffer from major shortcomings. First, long-term EEG is usually highly contaminated with artefacts. Second, changes in the EEG signal over long intervals, known as concept drift, are often neglected. We evaluate the influence of these problems on deep neural networks using EEG time series and on shallow neural networks using widely-used EEG features. Our patient-specific prediction models were tested in 1577 hours of continuous EEG, containing 91 seizures from 41 patients with temporal lobe epilepsy who were undergoing pre-surgical monitoring. Our results showed that cleaning EEG data, using a previously developed artefact removal method based on deep convolutional neural networks, improved prediction performance. We also found that retraining the models over time reduced false predictions. Furthermore, the results show that although deep neural networks processing EEG time series are less susceptible to false alarms, they may need more data to surpass feature-based methods. These findings highlight the importance of robust data denoising and periodic adaptation of seizure prediction models.

## Introduction

Epilepsy is a chronic neurological disease characterised by brief and recurrent episodes known as seizures^1,2^. It affects 1% of the world’s population. About one-third of people with epilepsy are diagnosed with drug-resistant epilepsy (DRE)^3^. DRE is diagnosed when at least two antiseizure drugs fail to lead the patient to a stable seizure-free condition^4^. This condition is often a severe limitation because patients are not allowed to perform regular daily tasks such as driving and usually have restrictions in their professional life. Additionally, since these patients continue to have seizures, there is a high risk of brain injuries, accidents, and even sudden unexpected deaths (SUDEP)^5^. Therefore, a warning device able to anticipate seizures could improve their lives. As soon as this warning device predicts an upcoming seizure, it raises an alarm, enabling the patient to take some measures to avoid accidents or even suppress the seizure by taking seizure-suppressing drugs^6–9^.

Seizure prediction has been an active research theme since 1970^10^. Epileptic electroencephalogram (EEG) is normally divided into four periods: preictal, ictal, postictal, and interictal. The preictal period corresponds to the interval before the seizure onset; the ictal period concerns the seizure; the postictal period is the period right after the ictal interval; and the interictal period is the seizure-free period between the postictal of the previous seizure and the preictal of the following one. The main goal of seizure prediction is to develop a system to anticipate upcoming seizures, i.e., to identify the transition from the interictal to the preictal period. Several studies have been published in this research area, typically based on electroencephalogram as it can record the electrical brain activity^11,12^. Initially, seizure prediction models were threshold-based, meaning that when a given EEG biomarker (feature) surpassed a pre-defined threshold, seizure alarms would be raised^13,14^. However, these models were linear and based on a single feature, which might not be sufficient to perceive the complexity of the pre-seizure activity^15,16^. Later, shallow machine learning algorithms were employed with acceptable results for some patients^12,17,18^. These algorithms could establish relations between different EEG biomarkers, improving the capability of the models to find pre-seizure patterns^19–25^. In recent years, deep learning architectures have been increasingly used in multiple research areas^26^. These architectures are not dependent on the computation of handcrafted features before classification, as they can extract information directly from the data, i.e., explore the patterns present in the data without a prior definition or equation (abstract features). Moreover, as these models are able to automatically extract and select the optimal features, less feature engineering and domain knowledge are needed to develop intelligent systems. Although these characteristics turn machine learning models into black-boxes, they could be advantageous when there is no solid physiological grounding. Therefore, authors have started to use them to develop seizure prediction approaches using EEG signals or multidimensional data computed from EEG signals^27–48^. Despite the ability to extract knowledge directly from the data, some researchers still extract features using traditional signal processing methods while using deep learning approaches^16,49–51^.

Even though the increasing complexity of seizure prediction algorithms is a significant topic, others are equally important. An example is the EEG preprocessing^18,52^. Researchers are moving towards non-invasive EEG to develop seizure prediction approaches^53^. However, these signals usually present artefacts. EEG artefacts may be responsible for the increase of false alarms and should be removed before creating the seizure prediction models^40,54,55^. Researchers mostly consider simple digital filters to remove unwanted frequency bands^47,48,56,57^, such as high-pass filters to remove the DC component and low-pass filters to diminish the influence of high-frequency noise. However, these filters suppress whole frequency bands and can not remove physiological artefacts, such as eye and muscle artefacts, which often overlap with important brain activity spectra^58,59^. Consequently, other techniques have been used to reduce the influence of this noise on seizure prediction. Myers et al.^60^ removed all segments containing artefacts. Despite being accurate in removing artefacts, this technique implies discarding a significant amount of EEG data. Bandarabadi et al.^21^ reduced the influence of the artefacts using a moving average to compute the features and a regularisation method on the classifier’s output. Also, Parvez et al.^61^ smoothed the classifier’s output to mitigate the effect of artefacts. Islam et al.^62^ used independent component analysis (ICA) to remove artefacts. Borhade et al.^51^ removed artefacts using a preprocessing module capable of separating them from the neural activity. Das et al.^63^ used wavelet decomposition to remove noise. Usman et al.^64^ employed empirical mode decomposition to reduce the influence of the artefacts. Prathaban et al.^41^ reported a custom method to reconstruct EEG signals based on sparsity. Although different methods were used, all the aforementioned authors reported that removing artefacts improved seizure prediction. However, none of the presented studies compared the prediction performance of models developed using noisy data with models developed using denoised data.

Typically, researchers train seizure prediction models using the first chronological seizures and evaluate them on the following seizures without considering concept drifts that occur over time. These changes in data distribution may occur as a result of the seizure events, an alteration of antiepileptic drug type and/or dosage, and biological cycles (e.g., circadian rhythms), which might alter the dynamics of the brain^65–71^. Dealing with concept drifts requires a different approach for training computational models. Several authors proposed solutions based on simply periodically refitting the models^25,37,70–72^. Kiral-Kornek et al.^72^, Nejedly et al.^37^, and Chen et al.^25^ used EEG data collected over several months. They retrained their models every month and eliminated past data after a certain amount of months, enabling the models to re-adapt over time. Pinto et al.^70,71^ used EEG signal collected under pre-surgical conditions. Therefore, they did not use data from several months, but only from a few days. They retrained their models after testing on a new seizure. Although those studies tried to deal with concept drifts, only Nejedly et al.^37^ verified that there was an improvement in the prediction performance.

The present study addresses some important aspects faced when developing seizure prediction models. We explored the effect of using a deep convolutional neural network-based EEG artefact removal model, able to mimic manual preprocessing made by experts, on the prediction performance. Furthermore, we evaluated the influence of retraining the models over time to handle possible concept drifts. Both comparisons were performed using a deep convolutional neural network connected to a bidirectional long short-term memory layer (CNN-BiLSTM) using EEG time series as input and a shallow artificial neural network trained using established handcrafted features. In summary, our study comprehensively assesses the impact of denoising and dealing with the presence of concept drifts in deep learning models fed with EEG time series and in handcrafted feature-based ones.

## Methods

### Database

In this study, we used data from the European Epilepsy Database, also known as the EPILEPSIAE database (www.epilepsy-database.eu), which was developed by the FP7 EPILEPSIAE project (www.epilepsiae.eu). The EPILEPSIAE database consists of long-term EEG signals acquired from 275 patients with epilepsy who underwent pre-surgical monitoring over several days^7^. The data were collected at Universitätsklinikum Freiburg (Germany), Centro Hospitalar e Universitário de Coimbra (Portugal), and Hôpital de la Pitié-Salpêtrière, Paris (France). The use of these data for research purposes has been authorised by the Ethical Committee of the three hospitals involved in the EPILEPSIAE database development (Ethik-Kommission der Albert-Ludwigs-Universität, Freiburg; Comité consultatif sur le traitement de l’information en matière de recherche dans le domaine de la santé, Pitié- Salpêtrière University Hospital; and Comité de Ética do Centro Hospitalar e Universitário de Coimbra). All studies were performed following relevant guidelines and regulations, and informed written consent was obtained from the patients.

For this study, we considered scalp EEG data collected from 41 patients (24 males, mean age: 41.41 ± 15.67 years) with temporal lobe epilepsy (TLE), the most common type of focal epilepsy^73^, at Universitätsklinikum Freiburg. The data were acquired using a sampling rate of 256 Hz and 19 electrodes placed according to the 10-20 international system. We selected only those patients who had at least three leading seizures^74^, separated by no less than 4 hours and 30 minutes. These data comprise approximately 5,600 hours of recording time containing 227 seizures. More details about the dataset can be found in Table S1, which is available in the supplementary material.

### Seizure prediction pipeline

**Figure 1:**
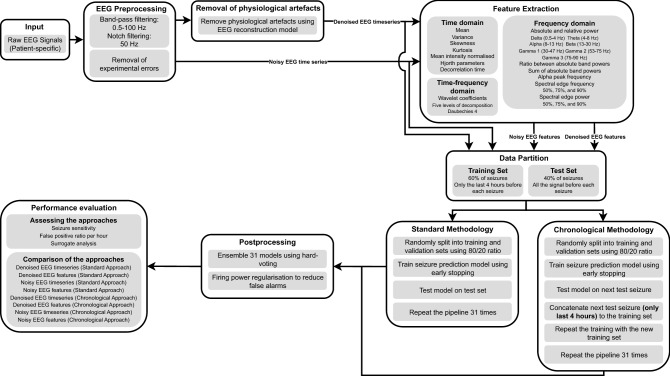
Seizure prediction pipeline comprising EEG preprocessing, feature extraction, data partition, training approaches, postprocessing, and evaluation procedure. All models were trained following a patient-specific approach. Therefore, this pipeline was repeated for each patient, individually.

The seizure prediction pipeline begins by preprocessing the EEG signals using digital frequency filters and removing experimental errors. Afterwards, the pipeline presents two branches: one in which the physiological artefacts are removed (**denoised EEG time series**), and another where they are not removed (**noisy EEG time series**). Then, we extracted features from the resulting datasets (**denoised EEG features** and **noisy EEG features**). EEG time series are used on models based on deep neural networks, whereas EEG features are used to develop shallow neural networks. Next, each dataset is similarly divided into training and test sets. The datasets are then used to develop seizure prediction models following two different approaches: the **standard approach**, which consists of training only once and testing on the remaining seizures, and the **chronological approach**, which involves retraining after every new test seizure. Subsequently, we performed postprocessing methods on the test set predictions and finally evaluated the performance of the approaches and compared them. Figure 1 illustrates the pipeline followed in this study. It is worth noting that this pipeline is performed individually for each patient as every model is patient-specific.

### Preprocessing

Signal preprocessing was performed using an algorithm presented in Lopes et al.^75^, which mimics the manual preprocessing made by experts. It is divided into three phases. Firstly, frequency filtering was performed using a 0.5–100 Hz fourth-order bandpass filter and a 50 Hz second-order notch filter. After that, the algorithm removed experimental errors, such as flatlines, saturated segments, and data with very high amplitude. Additionally, segments with too many artefacts were also removed. Afterwards, the artefact removal model based on deep convolutional neural networks (CNNs), developed by Lopes et al.^75^, was used to remove physiological artefacts such as eye, muscle, and cardiac artefacts. This model was developed using data from the EPILEPSIAE database. Subsequently, we discarded the first 30 minutes of the signal after each seizure onset to eliminate the influence of a possible postictal state^76^. Finally, the EEG segments were divided into 10-second windows. After the preprocessing methods, the data comprise approximately 4650 hours of recording time.

### Feature extraction

After preparing EEG data, we extracted established EEG features^18^ using signal processing methods. We used time-domain linear univariate features (mean, variance, skewness, kurtosis, mean intensity normalised, Hjorth parameters, and decorrelation time), frequency-domain linear univariate features (absolute and relative band powers of the following bands: 0.5–4 Hz (delta), 4–8 Hz (theta), 8–13 Hz (alpha), 13–30 Hz (beta), 30–47 Hz (gamma 1), 53–75 Hz (gamma 2), and 75-90 Hz (gamma 3); the ratio between every spectral band powers, the sum of all absolute band powers, the alpha peak frequency, and the spectral edge frequency and spectral edge power for 50%, 75%, and 90%), and time-frequency domain linear univariate features (wavelet coefficients computed using mother-wavelet Daubechies 4 with five levels of decomposition). These features were computed for every 10-second window and every channel. Only univariate linear features were considered due to their fast computation time.

### Seizure occurrence period and seizure prediction horizon

**Figure 2:**
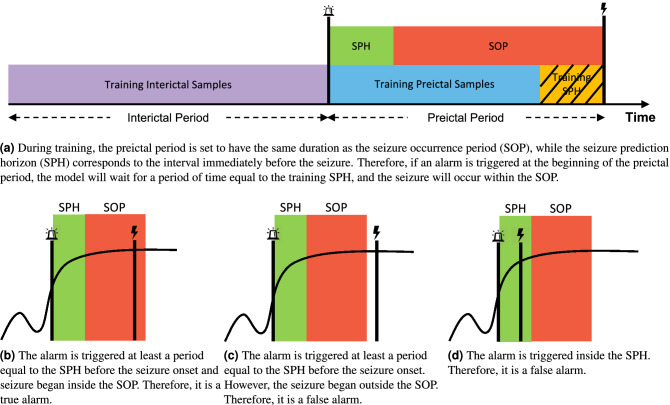
Representation of how to train a seizure prediction model and the requirements needed to be considered a true alarm.

Seizure occurrence period (SOP) and seizure prediction horizon (SPH) are fundamental for developing and assessing seizure prediction models. As presented in Fig. 2a, the SPH allows the model to provide the patient with a period of time to take countermeasures before a seizure, whereas the SOP is the period when the seizure occurs. During training, preictal samples, which are samples taken before the seizure, correspond to an interval with the same duration as the SOP. The samples following the training preictal samples and ending at the seizure’s onset correspond to the SPH and are not included in the analysis. This ensures that in the case of a true alarm, the patient will have an interval equal to the SPH to take countermeasures before the upcoming seizure, which is expected to occur within a period of time equal to the SOP.

Over the years, there has been no consensus on the most optimal SOP. Extensive research has been conducted to find it, using grid search^21,50,70,71^ or unsupervised procedures^77–79^. According to the aforementioned papers, the optimal SOP is typically between 30 and 60 minutes. Recently, researchers have been using an SOP of 30 minutes not only because it falls within the optimal range of SOPs observed in previous findings, but also because it is short enough to avoid causing anxiety in patients^29,34,38,39,43^. As a result, we used an SOP of 30 minutes in our study. The SPH was set to 10 minutes, allowing patients to take a seizure-suppression drug to prevent the seizure^80^. Accordingly, all samples located up to 40 minutes before the seizure onset were labelled as interictal (class 0). The samples corresponding to the training SOP were considered preictal (class 1). The samples during the training SPH were discarded. Figure 2b–d show a true alarm and two false alarms, respectively.

### Training and test sets

The training set was composed of 60% of the available data, and the remaining 40% was allocated to the test set. This division was performed chronologically using the first 60% of the seizures for training. Data preprocessing, as explained above, involved the removal of some data that could not be used. Therefore, there was insufficient preictal data for some seizures to be correctly predicted during testing. As a result, one test seizure from patient 52302 had to be removed, and both sets from that patient were updated to maintain the 60/40 ratio. Finally, to reduce the training computation time, we only used the four hours before each seizure’s onset during the training phase. In the case of the test set, for each seizure, it included all the data from 30 minutes after the previous seizure onset until the onset of the seizure under analysis. Ultimately, the training set contained 540 hours of EEG data and 135 seizures, whereas the test set comprised approximately 1577 hours of EEG data and 91 seizures. Details of the training and test sets can be found in Table S2 in the supplementary material.

### Artificial neural network architectures

**Figure 3:**
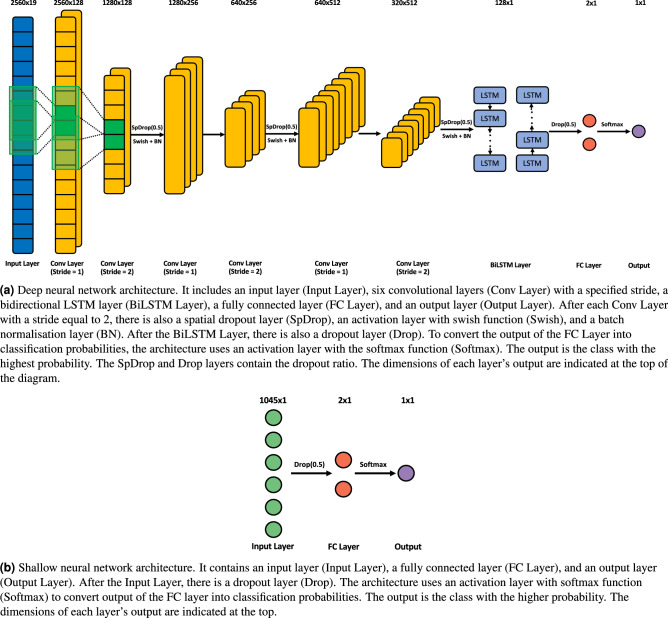
Neuronal network architectures used to develop seizure prediction models. (**a**) Deep neural network, which takes 10-second EEG time series as input. (**b**) Shallow neural network, which is based on EEG features.

As presented in Fig. 3, we used two distinct neural networks: a deep neural network, based on the CNN-BiLSTM architecture, which is capable of automatically processing EEG time series (**deep classifier**), and a shallow neural network based on fully connected (FC) layers with handcrafted features as input (**shallow classifier**).

The architecture presented in Fig. 3a is a CNN-BiLSTM model. It consists of three blocks of convolutional layers and a bidirectional LSTM layer. Each block contains two convolutional layers, one of which has a stride of 2 and is used as a learnable pooling layer. Additionally, each block contains a spatial dropout layer with a 50% rate, an activation layer with the swish function, and a batch normalisation layer. The swish function is described by Eq. 1 where *x* is the input vector.

1 $$\begin{aligned} f(x)=x\, \times \, sigmoid(x) \end{aligned}$$

The number of filters starts at 128 and doubles with every new block. The filter size for every layer is 3. After the convolutional blocks, the samples are processed by the bidirectional LSTM layers, each containing 64 units. A dropout layer with a 50% rate is then applied. Finally, the samples are classified using an FC layer with two neurons and an activation layer with the softmax function, which is described by Eq. 2, where *x* is the input vector.

2 $$\begin{aligned} f(x)=\frac{e^{x_i}}{\sum _{j}^{}e^{x_j}} \end{aligned}$$

The input dimension is 2560x19, which means that each sample consists of 10 seconds of EEG acquired at 256 Hz and 19 channels. All hyperparameter values were obtained from a grid search process. Specifically, the grid search for the deep classifier was performed to find the best hyperparameters out of the following ones:

- Number of filters of the first block: [32, 64, 128];
- Filter size: [3, 5, 7];
- LSTM units: [32, 64, 128].

The architecture presented in Fig. 3b consists of four layers: an input layer, a dropout layer with a 50% rate, an FC layer with two neurons, and an activation layer with the softmax function. The input dimension is 1045x1, which means that it comprises 55 features computed over 19 channels. The grid search for the shallow classifier was conducted to identify the optimal hyperparameters among the following:

- Number of neurons in the FC layer: [None, 8, 16, 32, 64, 128, 256].

No feature selection was performed before classification to enable the shallow neural network to determine which features could contribute more to the prediction performance. Both neural networks comprise dropout layers with a 50% rate, which was selected to address overfitting caused by the limited number of training samples. Both grid searches were conducted using the training set of ten randomly selected patients. To evaluate the hyperparameters, we used the data of the last seizure from the training set and computed the geometric mean of sensitivity and specificity. The grid search was repeated three times for each combination and each patient training set, and the results were averaged to compare the performance and select the best hyperparameters. The selected hyperparameters were used in all patient-specific models. Detailed results can be found in Tables S3 and S4 in the supplementary material.

### Training methodologies

We used two methodologies to train our patient-specific models: standard and chronological. In the standard training, seizure prediction models were trained using a static training set and tested on subsequent seizures. The chronological training involved training seizure prediction models using the first set of seizures, testing on the following seizure, concatenating it (EEG signal and labels) to the previous training set, and repeating the process until all seizures were tested. Data partitioning and standardisation were performed each time the training set was updated. We repeated both methodologies 31 times, resulting in 31 models that were used to perform a majority voting ensemble, whereby an odd number of models avoids ties. Furthermore, in a real-life scenario 31 different performances per patient are unfeasible. A majority voting ensemble helped to mitigate the variability of each trained seizure prediction model and produced a single model for each approach instead of 31 different models.

We trained the neural networks using batches of 64 samples, with each batch containing 32 interictal samples and 32 preictal samples. To address the imbalance between the classes, we oversampled the minority class by replicating preictal samples. We considered 500 training epochs and used early stopping regularisation with a patience of 50 epochs to prevent overfitting. We used the adaptive moment estimation (Adam)^81^ as the optimisation algorithm, with an initial learning rate of 3e$-4$. The loss function was binary cross-entropy. Early stopping requires a validation set to constantly verify whether the model is overfitting. Therefore, we randomly divided the training set into a new training set and a validation set according to an 80/20 ratio. In contrast to the data partition step, which was performed on a seizure level, the 80/20 division was performed on the samples. Training, validation, and test sets were normalised using z-score calculated based on the training samples. Table 1 provides a summary of the training settings.

**Table 1:** Hyperparameters used to train the neural networks.

Hyperparameter	Value
Dataset partition	Holdout validation 80/20
Optimisation function	Adam
Learning rate	3e$-$4
Loss function	Binary cross-entropy
Epochs	500
Patience epochs (early stopping)	50
Runs	31

### Postprocessing

Firing power regularisation^82^ was used to reduce the number of possible false alarms. The method consists of applying a moving window with a size equal to SOP, which accumulates the predicted output of several samples. The mathematical formulation of this regularisation method is given by

3 $$\begin{aligned} fp[n] = \frac{\sum _{k=n-\tau }^{n}o[k]}{\tau } \end{aligned}$$

where *fp*[*n*] is the firing power regularisation output, $\tau$ is the number of samples of the moving window, and *o*[*k*] is the seizure prediction model output at time *k*. An alarm is triggered as soon as the ratio of preictal instants in the moving window exceeds a threshold of 0.5^21,50^. After each alarm, we applied a refractory period^70,71^ of 40 minutes, which corresponds to the concatenation of the SPH and SOP intervals. During this period, the models could not raise any alarm. The refractory periods were implemented to prevent the patient from being overwhelmed with successive alarms in a short period of time. It is worth noting that our implementation of the firing power is an adaptation of the method proposed by Teixeira et al.^82^. We adapted the method to handle temporal gaps resulting from unconnected windows after preprocessing. Thus, when there is a gap, the firing power considers it as several windows with a null value, decreasing until reaching zero when the gap is too long.

### Performance evaluation

To evaluate the performance of the seizure prediction models, we used three metrics: seizure sensitivity, false alarm rate per hour (FPR/h), and the number of patients with performance above chance level through surrogate analysis. Seizure sensitivity and FPR/h were computed using the Eqs. 4 and 5.

Seizure sensitivity measures the ratio between the number of true alarms ($\#\,True\,Alarms$) and the number of testing seizures ($\#\,Test\,Seizures$).

4 $$\begin{aligned} Seizure\,Sensitivity = \frac{\#\,True\,Alarms}{\#\,Test\,Seizures} \end{aligned}$$

FPR/h is defined as the ratio between the number of false alarms ($\#\,False\,Alarms$) and the total duration of the interictal period ($Interictal_{Duration}$) without the periods right after false alarms when there could not be triggered any new alarm ($\#\,False\,Alarms\times Refractory_{Duration}$).

5 $$\begin{aligned} FPR/h = \frac{\#\,False\,Alarms}{Interictal_{Duration}-\#\,False\,Alarms\times Refractory_{Duration}} \end{aligned}$$

The surrogate analysis is based on the Monte Carlo method and consists of randomly shifting seizure times^70,71,83^. This method is used to verify if the models perform above chance level. Seizure prediction models are considered to perform above chance if their performances are higher than the surrogate performances with statistical significance, considering a significance level of 0.05.

We also performed pairwise hypothesis testing (with a significance level of 0.05) to compare the different approaches that were developed. These comparisons include:

- Understanding the effect of removing physiological artefacts on the seizure prediction models;
- Comparing standard training with retraining the models over time;
- Comparing deep neural networks using EEG time series with shallow neural networks using handcrafted EEG features.

### System specifications

All calculations were performed on a computer with two Intel Xeon Silver 4214 12-core 2.2 GHz, ten graphics (five NVIDIA Quadro RTX 5000 and five NVIDIA Quadro P5000), 768 GB of RAM, and Linux Ubuntu 16.04 LTS operating system. We developed the models using the Tensorflow 2.4.1 and Keras 2.4.3 libraries for Python 3.8.

## Results

We begin by analysing the results for each patient for every approach. Afterwards, we analyse the overall results and compare the approaches. To facilitate readability, we present the approaches in the following format:

- **Denoised EEG** $_{Standard}$: Deep neural network, with denoised EEG time series as input, trained using the standard procedure;
- **Denoised EEG** $_{Chronological}$: Deep neural network, with denoised EEG time series as input, trained using the chronological procedure;
- **Denoised Features** $_{Standard}$: Shallow neural network, with handcrafted EEG features, computed from denoised EEG time series, as input, trained using the standard procedure;
- **Denoised Features** $_{Chronological}$: Shallow neural network, with handcrafted EEG features, computed from denoised EEG time series, as input, trained using the chronological procedure;
- **Noisy EEG** $_{Standard}$: Deep neural network, with noisy EEG time series as input, trained using the standard procedure;
- **Noisy EEG** $_{Chronological}$: Deep neural network, with noisy EEG time series as input, trained using the chronological procedure;
- **Noisy Features** $_{Standard}$: Shallow neural network, with handcrafted EEG features, computed from noisy EEG time series, as input, trained using the standard procedure;
- **Noisy Features** $_{Chronological}$: Shallow neural network, with handcrafted EEG features, computed from noisy EEG time series, as input, trained using the chronological procedure.

### Individual performance of seizure prediction models

**Figure 4:**
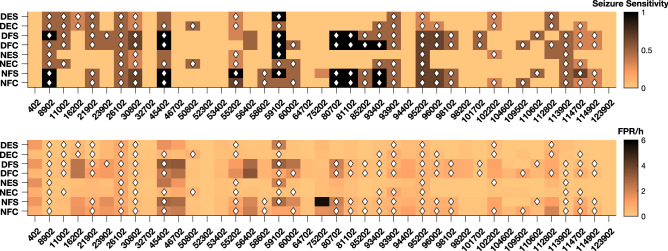
Results for each patient for DES (Denoised EEG$_{Standard}$), DEC (Denoised EEG$_{Chronological}$), DFS (Denoised Features$_{Standard}$), DFC (Denoised Features$_{Chronological}$), NES (Noisy EEG$_{Standard}$), NEC (Noisy EEG$_{Chronological}$), NFS (Noisy Features$_{Standard}$), and NFC (Noisy Features$_{Chronological}$) approaches. The top subfigure presents the seizure sensitivity obtained for each patient-specific model, while the bottom figure shows the FPR/h. The diamond symbol indicates that the model performed above chance level. The scales of the colours are on the right side of the subfigures.

Figure 4 shows the seizure sensitivities and the FPR/h of the patient-specific models evaluated on the test seizures of each patient. This figure also shows which models performed above chance level.

Inspection of the results obtained for each patient leads to the following conclusions:

- For several patient-specific models, all approaches performed similarly. For example, performance above chance level was obtained for all approaches for four patients (9.8%), whereas for twelve (29.3%), no approach performed above chance level.
- For three patients (7.3%), only models trained with denoised data performed above chance level.
- The transition from standard to chronological training decreased the number of false alarms for six patients (14.6%).
- For some patients, only one type of neural network obtained performance above chance level: Ten patients (24.4%) in the case of shallow neural networks using features and two patients (4.9%) in the case of deep neural networks.
- The Noisy Features$_{Standard}$ approach obtained a very high FPR/h for one patient (2.4%).

Detailed results are presented in Tables S5 and S6 in the supplementary material.

### Overall performance of seizure prediction models

**Figure 5:**
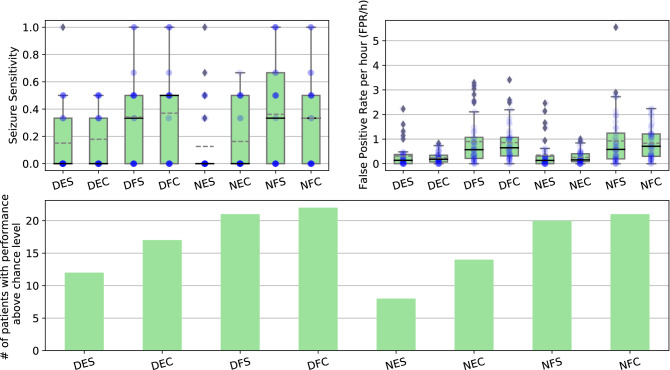
Boxplots with the overall seizure sensitivity and FPR/h for the DES (Denoised EEG$_{Standard}$), DEC (Denoised EEG$_{Chronological}$), DFS (Denoised Features$_{Standard}$), DFC (Denoised Features$_{Chronological}$), NES (Noisy EEG$_{Standard}$), NEC (Noisy EEG$_{Chronological}$), NFS (Noisy Features$_{Standard}$), and NFC (Noisy Features$_{Chronological}$) approaches. Continuous black lines represent medians, dashed grey lines correspond to the averages, diamonds symbolise outliers, and the distributions of the results for each patient are presented as blue circles. Bar charts show the number of patients’ models with performance over chance using surrogate analysis.

Table 2 summarises the overall results of all developed approaches. Boxplots with the overall seizure sensitivities and FPR/h for all approaches, as well as the distributions of the results, are displayed in Fig. 5. Additionally, it contains a bar chart with the number of patients with a performance above chance level for each approach. Table 3 presents the p-values obtained for pairwise statistical comparisons. Comparisons were made for seizure sensitivity and FPR/h metrics using one-tail Mann-Whitney U test^84^ considering an $\alpha$ value of 0.05.

**Table 2:** Average results of the seizure prediction models for all approaches, for all 41 patients.

Approach	Seizure sensitivity	FPR/h	Above chance level (%)
**Denoised EEG** $_{Standard}$	0.15±0.24	0.31±0.48	12 (0.29)
**Denoised EEG** $_{Chronological}$	0.18±0.22	0.24±0.23	17 (0.42)
**Denoised Features** $_{Standard}$	0.34±0.35	0.90±0.96	21 (0.51)
**Denoised Features** $_{Chronological}$	0.37±0.36	0.86±0.77	22 (0.54)
**Noisy EEG** $_{Standard}$	0.13±0.24	0.35±0.58	8 (0.20)
**Noisy EEG** $_{Chronological}$	0.16±0.23	0.25±0.26	14 (0.34)
**Noisy Features** $_{Standard}$	0.36±0.38	0.93±1.09	20 (0.49)
**Noisy Features** $_{Chronological}$	0.33±0.36	0.83±0.65	21 (0.51)

**Table 3:** *P* values obtained for the statistical comparisons performed between all developed approaches using seizure sensitivity and FPR/h values. The comparisons were performed using one-tail Mann-Whitney, considering an *alpha* of 0.05. For seizure sensitivity, the p-values correspond to the probability of the distribution of group B being greater than the distribution of group A. For FPR/h, the p-values correspond to the probability of the distribution of group B being lower than the distribution of group A, except for the comparison between deep neural networks (EEG time series) and shallow neural networks (EEG features). In this particular case, the FPR/h values obtained for group B were higher, so the p-values correspond to the probability of group B being greater than group A.

Approaches	Group A	Group B	*P* values	
			Seizure sensitivity	FPR/h
**Noisy EEG/Denoised EEG**	**Noisy EEG** $_{Standard}$	**Denoised EEG** $_{Standard}$	0.273	0.435
	**Noisy EEG** $_{Chronological}$	**Denoised EEG** $_{Chronological}$	0.349	0.533
	**Noisy Features** $_{Standard}$	**Denoised Features** $_{Standard}$	0.580	0.554
	**Noisy Features** $_{Chronological}$	**Denoised Features** $_{Chronological}$	0.327	0.465
**Standard training/Chronological training**	**Denoised EEG** $_{Standard}$	**Denoised EEG** $_{Chronological}$	0.213	0.746
	**Noisy EEG** $_{Standard}$	**Noisy EEG** $_{Chronological}$	0.183	0.678
	**Denoised Features** $_{Standard}$	**Denoised Features** $_{Chronological}$	0.360	0.682
	**Nois Features** $_{Standard}$	**Noisy Features** $_{Chronological}$	0.616	0.693
**Deep neural networks (EEG time series)/Shallow neural networks (EEG features)**	**Denoised EEG** $_{Standard}$	**Denoised Features** $_{Standard}$	**0.003**	${\textbf {< 0.001}}$
	**Denoised EEG** $_{Chronological}$	**Denoised Features** $_{Chronological}$	**0.005**	${\textbf {< 0.001}}$
	**Noisy EEG** $_{Standard}$	**Noisy Features** $_{Standard}$	**0.001**	${\textbf {< 0.001}}$
	**Noisy EEG** $_{Chronological}$	**Noisy Features** $_{Chronological}$	**0.012**	${\textbf {< 0.001}}$

Significant values are in bold.

Some approaches based on EEG time series results in seizure sensitivities with null medians (Fig. 5), as those approaches scored null seizure sensitivities for more than half of the patients.

With the exception of the Denoised Features$_{Standard}$ approach, all models developed with denoised data obtained higher average seizure sensitivities compared to those using noisy data. Furthermore, the average FPR/h values were mostly lower in models based on denoised data. However, these comparisons did not show statistically significant differences.

Except for the Noisy Features$_{Chronological}$ approach, all models developed following a chronological methodology performed higher average seizure sensitivities compared to the ones following the standard procedure. The average FPR/h values obtained for models based on the chronological methodology were lower than those for the standard training, whereas the medians showed the opposite behaviour. This was due to the high number of outliers occurring on the approaches based on standard training. Nevertheless, all comparisons did not yield statistically significant differences.

Models based on deep neural networks returned lower average seizure sensitivities than those obtained for the models based on shallow neural networks. However, these lower seizure sensitivities were combined with low FPR/h values, meaning that, on average, deep learning models were more conservative on triggering an alarm. All the comparisons yielded statistically significant differences for both metrics.

The number of patients with performance above chance level increased when shifting from noisy to denoised data and from the standard to chronological training. The increase was greater in deep neural networks using EEG time series compared to the shallow neural networks using handcrafted features. However, the shallow neural networks obtained a higher number of patients performing above chance level.

In addition to the machine learning architectures used in this study, we evaluated the effectiveness of denoising data and chronological training using algorithms presented by other researchers, including a CNN using spectrograms proposed by Truong et al.^30^ and a logistic regression using handcrafted features proposed by Karoly et al.^24^. We selected these architectures to verify whether our findings were observed in other types of classifiers. The results obtained using the model proposed by Truong et al.^30^ were similar to those obtained in our study. In the case of the model proposed by Karoly et al.^24^, we also found that using denoised data improved the performance. However, transitioning from standard to chronological training did not result in any improvement. The implementation details and results are described in Sect. 3.4 of the supplementary material.

## Discussion

We analysed the impact of two essential aspects for developing patient-specific seizure prediction models: denoising EEG signals and retraining the models over time. The prediction models were developed using deep neural networks with EEG time series as input and shallow neural networks using widely used EEG features.

The EEG artefact removal model was already proposed and evaluated in Lopes et al.^75^ regarding its capacity to reconstruct EEG signals. As a next step, we wanted to evaluate how far the artefact removal method can improve the results for seizure prediction. For most cases, using the artefact removal model to denoise EEG signals before developing the seizure prediction models resulted in an improvement in seizure sensitivities, FPR/h values and the number of patients with performance above chance level. In the case of deep neural networks, removing artefacts using the automatic denoising model led to a small reduction in the number of outliers regarding FPR/h. This was expected since artefacts can change signal characteristics and mask some patterns that could be important to assess seizure susceptibility^40,54,55^.

Concerning retraining over time, we observed different behaviours for different metrics. Seizure sensitivity did not always increase from standard to chronological training. However, FPR/h and the number of patients with a performance above chance level improved after retraining. Thus, we conclude that the models benefited from chronological training, either by having more training data available or by adapting to possible concept drifts that occur over time (see Fig. S1 of the supplementary material). This resulted in a reduction in the number of false alarms and an increase of the number of patients with performance above chance level^25,37,72^.

Comparing both model types, although deep learning models returned lower seizure sensitivities, they also yielded lower FPR/h values by being more conservative in firing alarms. The number of patients performing above chance level was also lower for the deep learning models, which is mainly attributed to their lower sensitivity. Models that did not predict any test seizure could not be validated using surrogate analysis, thus leading to a lower number of patients with performance above chance level.

It is worth noting that although both neural network types improved after denoising and chronological training, the improvement was more evident for deep learning models than for shallow neural networks. Deep neural networks are data-driven architectures^26^. Consequently, features are automatically extracted from the EEG time series. On the other hand, feature-based models are trained using values obtained from established equations based on research knowledge acquired over the years. For this reason, each retraining only adapts the model weights used for the classification. Therefore, deep learning architectures, adapting to the input training data distribution, may be more affected by the quantity and quality of input data and the temporal proximity to the next seizure.

After analysing the results, we compared them with other studies that used scalp EEG data from the EPILEPSIAE database to develop seizure prediction models^23,34,70,71^. In this paragraph, we focus on the ones for which we did not have access to patient identification^23,34^. Direito et al.^23^ applied a simple preprocessing method using digital filtering and developed a seizure prediction model based on multiclass SVM using handcrafted features. They used firing power regularisation with a threshold of 0.5 to smooth the output of the classifiers. They considered a set of SOPs between 10 and 40 minutes and an SPH of only 10 seconds. They reported a seizure sensitivity of 39% and an average FPR/h of 0.21. The percentage of patients performing above chance level was approximately 10%. Nevertheless, it is worth noting that they validated their models using the analytical random predictor^85^ instead of the surrogate analysis. Although their seizure sensitivity and FPR/h are better than ours, it should be noted that they considered an SPH of just 10 seconds, which may not be sufficient time for a patient to take countermeasures before an upcoming seizure. Truong et al.^34^ also performed a simple preprocessing method using digital filtering. They developed a generative adversarial network using EEG time series as input and considered an SOP of 30 minutes and an SPH of 5 minutes. They reported an average AUC of 0.65 for 30 patients and performance above chance level for 23 patients using the Hanley-McNeil AUC Test to compare AUC scores with an AUC of 0.5. Since the authors did not make these metrics available, we can not compare their study with ours regarding seizure sensitivity and FPR/h. In terms of the number of patients with performance above chance level, they obtained a better result than ours. However, it should be pointed out that they did not use the same statistical evaluation method, which precludes fair comparisons.

Concerning the studies with detailed patient information, Pinto et al.^70,71^ published two papers with seizure prediction models based on evolutionary algorithms, with a different number of patients analysed in each one. In both studies, the algorithms were trained following a chronological approach. In this paper, we used data from some patients who were also included in both Pinto et al. studies. In both of their experiments the preprocessing was simple, using only digital filters. In the first study^70^, they used data from 19 patients and obtained an average seizure sensitivity of 0.38 ± 0.19 and an average FPR/h of 1.03 ± 0.84, using an SOP of 30 minutes and an SPH of 10 minutes. Performance above chance level was obtained for 32% of the patients. In the second study^71^, they used data from 93 patients and obtained an average seizure sensitivity of 0.16 ± 0.11 and an average FPR/h of 0.21 ± 0.08 using a set of SOPs between 20 and 50 minutes and a SPH of 10 minutes. 32% of the patients obtained performance above chance level. Both studies used firing power regularisation with a threshold of 0.7. When analysing individual patients, we found twelve patients (29.3%) that performed above chance in both Pinto et al. studies and some of our approaches. These patients should be selected to further explore preictal changes, as different methods performed similarly. Our approaches and Pinto et al. models did not perform above chance level for six patients (14.6%). Common failed predictions could mean no preictal changes at least 10 minutes before the onset of any tested seizures. Pinto et al. obtained performance above chance for four patients (9.8%) in at least one of their studies, whereas none of our approaches performed above chance level for them. Also, there were thirteen patients (31.7%) for whom we obtained models performing above chance level, whereas none of Pinto et al. studies obtained significant results. These differences could be related to data preparation details or the type of algorithm used. In any case, the results of the seizure prediction studies seem to be coherent regarding the obtained statistics, even if the approaches were different, which supports the statements reported by Müller et al.^86^ about the high number of false positives generated by different types of seizure prediction models. Detailed results obtained by Pinto et al.^70,71^ are presented in Table S7 in the supplementary material.

Our study has some limitations that should be highlighted. The first one concerns the use of EEG signals acquired in pre-surgical conditions. Pre-surgical conditions involve drug type and/or dosage alteration and possible sleep deprivation as part of the clinical evaluation process, which may cause more concept drifts than expected. Additionally, if the signals are acquired outside the hospital for months, they may contain even more artefacts than the ones seen in the analysed data because there are no technicians to constantly check the equipment. Therefore, pre-surgical data do not fully simulate the daily behaviour of the patients^15^, and care should be taken before generalising these results to real-life situations.

Another limitation is the number of seizures per patient. The average number of total seizures per patient was 5.51, whereas the average number of tested seizures was 2.21. The low number of seizures limited the evaluation of the approaches since some patients had only one testing seizure restricting the obtained seizure sensitivity to 0.00 or 1.00. This large difference in possible seizure sensitivities produced large standard deviations which may have restricted the results of the statistical comparisons. Furthermore, as the amount of data was low, the improvement obtained by training periodically may have been due to the increase in available information rather than the change in concept. Thus, a higher number of seizures would allow for a better evaluation of the effect of retraining over time or even to test other different approaches to handle concept drifts^87^.

The seizures used in this study were manually annotated by experts. In real-time scenarios, manual annotation of seizures can be challenging since it is difficult for physicians to review all the acquired EEG signals. The solution to this restriction could be the one presented by Pal Attia et al.^88^, where data would be sent to a cloud along with the outputs of a seizure prediction model, a seizure detection model, and annotations sent by the patient. In this way, the technicians could quickly review only the events that were noted and eliminate all those that were not seizures. Then, the model could be retrained using data from the new seizure events.

The fixed duration of the SOP for all patients was also a constraint. In our case, we used a fixed SOP of 30 minutes for all patients, which is in line with the SOP duration used in other studies^21,29,34,38,39,43,50^. SOP and SPH determine the considered preictal samples. Therefore, we also limited the considered preictal interval. The preictal interval of seizures could last from few minutes to several hours. Thus, EEG characteristics should be carefully inspected in order to not consider preictal patterns as interictal data^78,79^. However, the inspection of the optimal SOP was not in the focus of this study.

The fifth limitation was the number of hours used to train our prediction models. In our case, we only used four hours per seizure to train the models. We selected this time to overcome the high imbalance between interictal and preictal periods as well as to reduce computation time. However, it could limit the training of the models because they might not be able to learn critical long-term patterns such as the circadian cycle^65,66^. Lastly, our models are trained to trigger alarms once they detect any preictal changes in the data. Therefore, they can not measure seizure susceptibility over time and decide if one alarm is more important than others. Additionally, the brain has its own regulatory system. There may be some scenarios in which the model predicts a seizure due to the high seizure susceptibility state, but the brain triggers seizure control mechanisms before reaching the ”point of no return”^89,90^. This new perspective has prompted the change from developing seizure prediction models to designing seizure forecasting frameworks. The latter allows researchers to better understand what is happening in the brain because forecasting approaches output seizure risk instead of just alarms. Furthermore, forecasting models are generally less penalised during evaluation since their response is continuous and not based on ”all-or-nothing”. For example, in our approaches, if an alarm were raised 41 minutes before the seizure onset, it would be evaluated as a false alarm even if it was essentially a correct prediction^53^.

This paper explores two essential aspects that should be taken into consideration before developing seizure prediction models: the impact of performing a robust preprocessing to remove noisy artefacts, such as ocular artefacts, from EEG signals; and the importance of periodically retraining the seizure prediction models to address the possible presence of concept drifts. We investigated the importance of these two variables using two models: one based on deep neural networks with EEG time series as input and another based on shallow neural networks using handcrafted EEG features computed using signal processing techniques. The results evidenced that the performance of deep learning approaches improved after denoising the data and periodically retraining the models. On shallow neural networks with handcrafted features as input, the effect of denoising and/or retraining was barely noticeable, which may indicate that handcrafted features were more robust to changes in the data. The results also showed that shallow neural networks using handcrafted features were able to predict twice as many seizures as deep learning models. However, the number of false alarms was generally approximately four times higher as compared to deep neural networks. Therefore, when comparing both types of models, we can not conclude which performs better. As future work, these approaches should be tested with more patients and prospective data. Furthermore, it would be beneficial to test these approaches with more test seizures and with data collected over longer periods than just a few days, such as signals obtained by ultra long-term acquisition systems^69^.

## Supplementary Information

Supplementary Information.

## Data Availability

The data supporting the findings of this study are not publicly available due to restrictions from the EPILEPSIAE Consortium, who provided the data under license for this study. However, the data can be made available from the authors upon reasonable request and with permission from the EPILEPSIAE Consortium. The code used in this study is available for public use at https://github.com/fabioacl/Seizure-Prediction---Artifact-Removal-and-Chronological-Training.
