# Supplementary material for: Removing artefacts and periodically retraining improve performance of neural network-based seizure prediction models
Source: Sci Rep. 2023 Apr 11;13:5918. doi: 10.1038/s41598-023-30864-w (PMC10090199; doi:10.1038/s41598-023-30864-w)
Supplement: Supplementary file 1 — Supplementary Information. [file 41598_2023_30864_MOESM1_ESM.pdf]

Supplementary material for:

Removing artefacts and periodically retraining improve performance of neural network-based seizure prediction models

Fábio Lopes, Adriana Leal, Mauro F. Pinto, António Dourado, Andreas Schulze-Bonhage, Matthias Dümpelmann, and César Teixeira

1 Patient and seizure metadata

Table S1 contains information regarding the group of patients with temporal lobe drug-resistant epilepsy analysed in this study. The table includes information on sex, age at hospital admission and onset age (corresponding to the occurrence of the first epilepsy event), epilepsy foci lateralisation, the total number of annotated seizures and the number of lead seizures analysed for each patient, according to the considered minimum interseizure interval of 4.5 hours. The duration of the used EEG data regarding training seizures and testing seizures is also presented. It is worth noting that although we consider lead seizures to happen at least 4.5 hours after the previous one, we used only 4 hours for training.

Table S2 contains a description of data collected for each analysed seizure. It includes information about the EEG onset time, used interseizure time, vigilance state at the time of the seizure onset, seizure classification, and seizure activity pattern. Seizures were classified according to the International League Against Epilepsy (ILAE) classification[1]. The vigilance state corresponds to one of the following states of alertness and responsiveness: wakefulness, non-rapid eye movement sleep (NREM sleep, further subdivided into three sleep stages N1–3) and rapid eye movement (REM) sleep[2].

Table S1: Dataset description regarding each patient.

P	ID	Sex	Onset Age (years)	Admission Age (years)	Lat.	#Sz	#LSz	Training time (dd hh/mm/ss)	Testing time (dd hh/mm/ss)
1	402	F	10	55	L, R	5	5	00 12:00:00	01 05:43:57
2	8902	F	23	67	L	5	5	00 12:00:00	00 22:33:21
3	11 002	M	21	41	R	8	4	00 08:00:00	00 19:51:27
4	16 202	F	43	46	L, R	8	7	00 16:00:00	00 21:20:11
5	21 902	M	44	47	L	6	4	00 08:00:00	00 17:40:40
6	23 902	M	36	36	L	5	5	00 12:00:00	01 09:59:10
7	26 102	M	15	65	L	8	4	00 08:00:00	01 21:24:37
8	30 802	M	28	28	L, R	9	8	00 20:00:00	01 19:55:36
9	32 702	F	33	62	L, R	6	5	00 12:00:00	00 22:15:49
10	45 402	F	13	41	L, R	5	4	00 08:00:00	01 04:34:30
11	46 702	F	13	15	R	5	5	00 12:00:00	00 12:39:01
12	50 802	M	2	43	L	5	5	00 12:00:00	01 11:39:33
13	52 302	F	13	61	L	7	3	00 08:00:00	00 09:33:47
14	53 402	M	0	39	L, R	8	4	00 08:00:00	02 02:30:52
15	55 202	F	3	17	R, B	9	8	00 20:00:00	01 18:54:12
16	56 402	M	18	47	L, R	7	4	00 08:00:00	06 04:28:44
17	58 602	M	17	32	L	22	6	00 16:00:00	00 10:46:12
18	59 102	M	17	47	R	7	5	00 12:00:00	03 10:15:50
19	60 002	M	47	55	L, R	8	6	00 16:00:00	05 15:37:09
20	64 702	M	3	51	R	6	5	00 12:00:00	01 07:41:43
21	75 202	M	10	13	R	8	7	00 16:00:00	01 07:21:15
22	80 702	F	14	22	B	10	6	00 16:00:00	00 19:26:12
23	81 102	F	14	22	B	13	3	00 08:00:00	00 23:26:03
24	85 202	F	4	54	L	10	5	00 12:00:00	00 20:27:13
25	93 402	M	40	67	L	7	5	00 12:00:00	02 06:06:02
26	93 902	M	43	50	R	9	6	00 16:00:00	00 13:32:53
27	94 402	F	29	37	R	11	7	00 16:00:00	00 23:44:49
28	95 202	F	13	50	L	14	7	00 16:00:00	03 06:16:09
29	96 002	M	21	58	L, R	9	7	00 16:00:00	03 04:32:26
30	98 102	M	2	36	L	5	5	00 12:00:00	01 21:48:06
31	98 202	M	3	39	R	10	8	00 20:00:00	01 07:41:52
32	101 702	M	44	52	L, R	6	5	00 12:00:00	00 23:52:54
33	102 202	M	0	17	L	28	7	00 16:00:00	01 18:32:49
34	104 602	F	8	17	L	5	5	00 12:00:00	00 15:19:10
35	109 502	M	40	50	L, R	10	4	00 08:00:00	01 23:09:24
36	110 602	M	6	56	R	8	5	00 12:00:00	01 01:58:25
37	112 802	M	47	52	L	6	6	00 16:00:00	02 00:46:59
38	113 902	F	16	29	R	25	6	00 16:00:00	00 14:47:58
39	114 702	F	31	22	R	25	8	00 20:00:00	01 00:49:43
40	114 902	F	15	16	L, R	12	7	00 16:00:00	01 16:58:57
41	123 902	F	7	25	L, R	8	5	00 12:00:00	01 06:12:37

ID: patient identifier. Sex: female (F) or male (M). Lateralisation (Lat.): L: left, R: right, B: bilateral. #Sz: total number of seizures annotated per patient. #LSz: number of leading seizures, obtained as a result of the analysis of 4.5 hours of interseizure data.

Table S2: Dataset description regarding data preceding each seizure.

S	ID	EEG onset time	Used interseizure time (dd hh/mm/ss)	Vigilance state	ILAE Classification	Activity pattern
1	402	22:45:26	00 04:00:00	W	FOIA	t
2	402	21:27:34	00 04:00:00	W	FBTC	t
3	402	02:13:30	00 04:00:00	W	FOIA	t
4	402	08:53:21	00 06:09:51	W	FBTC	t
5	402	08:57:27	00 23:34:06	W	FOIA	t
6	8902	23:51:14	00 04:00:00	W	UC	a
7	8902	23:03:23	00 04:00:00	W	FOIA	b
8	8902	05:37:05	00 04:00:00	W	FOIA	a
9	8902	00:35:56	00 18:28:50	W	FOIA	m
10	8902	05:10:26	00 04:04:30	W	FOIA	a
11	11002	00:00:10	00 04:00:00	W	UC	?
12	11002	06:38:01	00 04:00:00	R	FOIA	s
13	11002	15:16:42	00 08:08:40	W	FOIA	a
14	11002	08:18:49	00 11:42:46	W	FOIA	t
15	16202	04:34:07	00 04:00:00	W	UC	r
16	16202	06:05:10	00 04:00:00	W	FBTC	?
17	16202	05:07:14	00 04:00:00	W	UC	r
18	16202	18:48:33	00 04:00:00	W	FOIA	r
19	16202	03:34:35	00 08:16:01	W	FOIA	r
20	16202	13:50:31	00 07:57:01	W	FOIA	?
21	16202	19:27:39	00 05:07:08	W	FOIA	r
22	21902	16:16:43	00 04:00:00	W	UC	t
23	21902	08:40:51	00 04:00:00	W	FOIA	t
24	21902	20:32:56	00 07:53:23	W	FOIA	t
25	21902	06:50:12	00 09:47:15	R	FOIA	b
26	23902	10:18:13	00 04:00:00	W	FOA	t
27	23902	20:50:38	00 04:00:00	W	FOA	t
28	23902	11:18:12	00 04:00:00	W	FOA	t
29	23902	16:48:02	01 04:59:49	W	FOA	d
30	23902	22:17:22	00 04:59:20	W	FOA	t
31	26102	15:31:37	00 04:00:00	W	FOIA	m
32	26102	08:33:50	00 04:00:00	W	FOIA	t
33	26102	07:52:54	00 22:49:04	W	FOIA	t
34	26102	11:36:45	00 22:35:32	W	FOIA	t
35	30802	04:33:31	00 04:00:00	R	FOA	t
36	30802	04:52:24	00 04:00:00	W	FOA	t
37	30802	10:58:12	00 04:00:00	N2	FOA	t
38	30802	22:58:11	00 04:00:00	W	FOA	t
39	30802	05:49:34	00 04:00:00	W	FOA	t
40	30802	02:48:42	00 20:29:08	R	FOA	t
41	30802	07:48:06	00 04:29:24	N2	FOA	t
42	30802	03:15:10	00 18:57:04	N2	FOA	t
43	32702	08:25:28	00 04:00:00	W	FOIA	t
44	32702	10:22:47	00 04:00:00	W	FOIA	t
45	32702	10:13:13	00 04:00:00	W	FOIA	t
46	32702	17:03:16	00 06:20:03	W	FOIA	r

S: seizure. ID: patient identifier. Seizure vigilance state: wakefulness (W), NREM sleep stage I (N1), NREM sleep stage II (N2), REM sleep stage (R). Seizure ILAE classification: focal onset aware (FOA), focal onset impaired awareness (FOIA), focal to bilateral tonic-clonic (FBTC), unclassified (UC). Seizure activity pattern: rhythmic alpha waves (a), rhythmic beta waves (b), cessation of interictal activity (c), rhythmic delta waves (d), amplitude depression (m), repetitive spiking (r), rhythmic sharp waves (s), rhythmic theta waves (t), unclear (?). Training seizures are highlighted in grey.

Continued on next page

S	ID	EEG onset time	Used interseizure time (dd hh/mm/ss)	Vigilance state	ILAE Classification	Activity pattern
47	32702	09:29:02	00 15:55:46	W	FOIA	a
48	45402	01:48:55	00 04:00:00	W	FOIA	t
49	45402	08:11:29	00 04:00:00	W	FOIA	t
50	45402	14:56:37	00 06:15:07	W	FOA	t
51	45402	15:13:34	00 22:19:21	W	FOIA	t
52	46702	15:56:40	00 04:00:00	W	FOA	a
53	46702	06:16:40	00 04:00:00	N2	FOIA	a
54	46702	17:06:57	00 04:00:00	W	FOIA	t
55	46702	02:02:23	00 08:25:26	N2	FBTC	b
56	46702	06:45:59	00 04:13:35	W	FOIA	t
57	50802	02:44:39	00 04:00:00	W	FOIA	t
58	50802	06:37:35	00 04:00:00	N2	UC	t
59	50802	12:39:04	00 04:00:00	N2	UC	t
60	50802	22:50:41	00 09:41:37	N2	FOIA	t
61	50802	01:18:38	01 01:57:56	W	FBTC	t
62	52302	06:29:39	00 04:00:00	W	UC	?
63	52302	11:31:13	00 04:00:00	W	FOA	?
64	52302	02:31:34	00 09:33:47	N1	UC	d
65	53402	08:16:32	00 04:00:00	W	FOA	?
66	53402	05:46:33	00 04:00:00	N2	FOA	?
67	53402	19:02:38	01 12:46:05	W	FOA	?
68	53402	09:17:43	00 13:44:46	W	FOIA	t
69	55202	07:02:49	00 04:00:00	W	FOIA	t
70	55202	09:55:11	00 04:00:00	W	FOIA	d
71	55202	18:15:11	00 04:00:00	W	FOA	t
72	55202	08:09:27	00 04:00:00	W	UC	t
73	55202	17:47:47	00 04:00:00	W	UC	t
74	55202	09:57:39	00 15:39:52	W	FOA	t
75	55202	15:34:54	00 05:07:15	W	UC	r
76	55202	14:11:59	00 22:07:05	W	FOIA	r
77	56402	08:17:30	00 04:00:00	W	UC	t
78	56402	21:11:53	00 04:00:00	W	UC	?
79	56402	09:13:46	05 07:13:23	W	UC	?
80	56402	06:29:39	00 20:15:19	W	FBTC	a
81	58602	09:11:25	00 04:00:00	W	FOIA	r
82	58602	03:29:21	00 04:00:00	R	FOIA	t
83	58602	19:52:52	00 04:00:00	W	FOIA	t
84	58602	09:01:07	00 04:00:00	W	FOIA	r
85	58602	15:41:02	00 04:50:43	W	FOIA	r
86	58602	02:31:58	00 05:55:28	N2	FOIA	t
87	59102	08:54:51	00 04:00:00	W	FOA	?
88	59102	15:41:55	00 04:00:00	W	FOIA	t
89	59102	09:56:35	00 04:00:00	W	FOIA	t
90	59102	19:51:41	00 09:25:05	W	FOIA	t
91	59102	21:12:26	03 00:50:44	W	FOA	t
92	60002	02:45:01	00 04:00:00	N1	FOIA	d
93	60002	02:22:55	00 04:00:00	W	FOIA	c

S: seizure. ID: patient identifier. Seizure vigilance state: wakefulness (W), NREM sleep stage I (N1), NREM sleep stage II (N2), REM sleep stage (R). Seizure ILAE classification: focal onset aware (FOA), focal onset impaired awareness (FOIA), focal to bilateral tonic-clonic (FBTC), unclassified (UC). Seizure activity pattern: rhythmic alpha waves (a), rhythmic beta waves (b), cessation of interictal activity (c), rhythmic delta waves (d), amplitude depression (m), repetitive spiking (r), rhythmic sharp waves (s), rhythmic theta waves (t), unclear (?). Training seizures are highlighted in grey.

Continued on next page

S	ID	EEG onset time	Used interseizure time (dd hh/mm/ss)	Vigilance state	ILAE Classification	Activity pattern
94	60 002	12:21:36	00 04:00:00	W	FOIA	t
95	60 002	05:40:53	00 04:00:00	R	UC	t
96	60 002	00:17:54	00 18:07:01	R	FOIA	d
97	60 002	22:18:46	04 21:30:51	N1	FOIA	d
98	64 702	13:53:39	00 04:00:00	W	FOA	?
99	64 702	04:23:21	00 04:00:00	W	FBTC	m
100	64 702	18:59:43	00 04:00:00	W	FBTC	t
101	64 702	19:50:01	01 00:20:17	W	FBTC	t
102	64 702	03:41:27	00 07:21:26	N2	FBTC	t
103	75 202	23:37:38	00 04:00:00	N2	FOA	t
104	75 202	01:10:45	00 04:00:00	N2	FOA	t
105	75 202	21:33:44	00 04:00:00	W	UC	t
106	75 202	19:27:00	00 04:00:00	W	FOA	t
107	75 202	09:46:19	00 13:49:19	W	FOA	t
108	75 202	17:43:46	00 05:20:22	W	FOA	?
109	75 202	06:25:19	00 12:11:33	W	FOA	t
110	80 702	05:03:56	00 04:00:00	W	FOIA	b
111	80 702	08:43:22	00 04:00:00	W	FOIA	b
112	80 702	20:43:38	00 04:00:00	W	UC	?
113	80 702	07:46:14	00 04:00:00	W	FOIA	c
114	80 702	17:54:17	00 04:56:33	W	FBTC	c
115	80 702	08:53:56	00 14:29:38	W	FOIA	c
116	81 102	20:48:50	00 04:00:00	W	FOIA	t
117	81 102	10:44:57	00 04:00:00	W	FOA	t
118	81 102	10:42:15	00 23:26:03	W	FOIA	t
119	85 202	23:37:05	00 04:00:00	N2	FOIA	m
120	85 202	16:51:04	00 04:00:00	W	FOIA	c
121	85 202	04:24:27	00 04:00:00	W	UC	m
122	85 202	16:08:00	00 11:13:33	W	UC	m
123	85 202	01:51:40	00 09:13:39	W	UC	m
124	93 402	22:17:50	00 04:00:00	N2	FBTC	t
125	93 402	10:21:34	00 04:00:00	N2	FOIA	t
126	93 402	23:20:24	00 04:00:00	N2	FOIA	t
127	93 402	00:59:09	02 01:08:44	N2	UC	t
128	93 402	06:26:26	00 04:57:17	N2	UC	t
129	93 902	08:39:52	00 04:00:00	W	FOA	t
130	93 902	16:02:21	00 04:00:00	W	FOIA	t
131	93 902	02:31:07	00 04:00:00	N2	FBTC	d
132	93 902	18:48:40	00 04:00:00	W	FOIA	d
133	93 902	04:02:38	00 08:43:58	N2	FOIA	d
134	93 902	09:21:33	00 04:48:55	W	UC	d
135	94 402	15:29:22	00 04:00:00	W	FOA	?
136	94 402	11:02:56	00 04:00:00	W	UC	d
137	94 402	18:05:40	00 04:00:00	W	FOIA	b
138	94 402	01:36:02	00 04:00:00	N2	UC	t
139	94 402	16:10:53	00 08:39:10	W	FOA	?
140	94 402	02:48:18	00 10:07:25	N2	UC	b

S: seizure. ID: patient identifier. Seizure vigilance state: wakefulness (W), NREM sleep stage I (N1), NREM sleep stage II (N2), REM sleep stage (R). Seizure ILAE classification: focal onset aware (FOA), focal onset impaired awareness (FOIA), focal to bilateral tonic-clonic (FBTC), unclassified (UC). Seizure activity pattern: rhythmic alpha waves (a), rhythmic beta waves (b), cessation of interictal activity (c), rhythmic delta waves (d), amplitude depression (m), repetitive spiking (r), rhythmic sharp waves (s), rhythmic theta waves (t), unclear (?). Training seizures are highlighted in grey.

Continued on next page

S	ID	EEG onset time	Used interseizure time (dd hh/mm/ss)	Vigilance state	ILAE Classification	Activity pattern
141	94402	08:16:30	00 04:58:11	W	FOA	?
142	95202	01:28:09	00 04:00:00	N2	FBTC	b
143	95202	15:00:18	00 04:00:00	N2	FOIA	b
144	95202	01:35:24	00 04:00:00	N2	FOIA	b
145	95202	14:13:22	00 04:00:00	N2	FOIA	m
146	95202	23:30:29	00 08:47:06	N2	UC	b
147	95202	23:55:21	00 23:54:52	N2	FOIA	b
148	95202	00:04:20	01 21:34:10	N2	UC	t
149	96002	17:10:35	00 04:00:00	W	FOIA	t
150	96002	10:26:53	00 04:00:00	W	FOIA	t
151	96002	17:46:44	00 04:00:00	W	FOIA	t
152	96002	00:05:44	00 04:00:00	W	FOIA	d
153	96002	00:44:10	00 23:56:34	W	UC	a
154	96002	18:57:18	00 17:43:07	W	FOIA	t
155	96002	06:20:01	01 10:52:43	W	FOIA	a
156	98102	07:17:49	00 04:00:00	W	FOA	?
157	98102	18:49:53	00 04:00:00	W	UC	?
158	98102	05:18:58	00 04:00:00	W	UC	?
159	98102	06:11:33	01 00:22:35	W	UC	?
160	98102	04:07:04	00 21:25:31	W	FBTC	?
161	98202	04:50:27	00 04:00:00	W	FOIA	t
162	98202	20:38:46	00 04:00:00	W	FOIA	a
163	98202	07:16:40	00 04:00:00	W	FOIA	t
164	98202	12:16:11	00 04:00:00	W	FBTC	t
165	98202	03:37:11	00 04:00:00	W	FOIA	t
166	98202	01:22:11	00 21:15:00	W	FOIA	t
167	98202	07:55:06	00 06:02:55	W	FOIA	t
168	98202	16:57:19	00 04:23:56	W	UC	t
169	101702	07:35:40	00 04:00:00	W	FOIA	t
170	101702	12:29:53	00 04:00:00	W	FOIA	t
171	101702	19:33:06	00 04:00:00	W	FOIA	t
172	101702	07:35:22	00 11:32:16	N2	FOIA	r
173	101702	20:26:01	00 12:20:38	W	FOIA	r
174	102202	22:50:21	00 04:00:00	N2	FOA	b
175	102202	15:36:30	00 04:00:00	W	UC	?
176	102202	05:47:03	00 04:00:00	N2	FOIA	t
177	102202	22:14:59	00 04:00:00	W	UC	?
178	102202	14:07:10	00 15:22:10	W	FOA	t
179	102202	06:16:20	00 09:47:18	N2	FOIA	t
180	102202	15:54:20	00 17:23:19	W	UC	t
181	104602	15:35:45	00 04:00:00	W	FOIA	t
182	104602	23:46:07	00 04:00:00	N2	FBTC	a
183	104602	06:24:56	00 04:00:00	N2	FBTC	t
184	104602	12:30:01	00 05:35:04	N2	FBTC	t
185	104602	22:44:07	00 09:44:06	N2	UC	d
186	109502	10:00:00	00 04:00:00	W	FOIA	t
187	109502	19:42:33	00 04:00:00	W	FOIA	t

S: seizure. ID: patient identifier. Seizure vigilance state: wakefulness (W), NREM sleep stage I (N1), NREM sleep stage II (N2), REM sleep stage (R). Seizure ILAE classification: focal onset aware (FOA), focal onset impaired awareness (FOIA), focal to bilateral tonic-clonic (FBTC), unclassified (UC). Seizure activity pattern: rhythmic alpha waves (a), rhythmic beta waves (b), cessation of interictal activity (c), rhythmic delta waves (d), amplitude depression (m), repetitive spiking (r), rhythmic sharp waves (s), rhythmic theta waves (t), unclear (?). Training seizures are highlighted in grey.

Continued on next page

S	ID	EEG onset time	Used interseizure time (dd hh/mm/ss)	Vigilance state	ILAE Classification	Activity pattern
188	109502	07:56:09	00 05:08:49	W	UC	t
189	109502	10:17:37	01 18:00:34	W	UC	t
190	110602	10:20:41	00 04:00:00	W	FOIA	t
191	110602	17:39:56	00 04:00:00	W	FOIA	t
192	110602	08:30:09	00 04:00:00	W	FOIA	t
193	110602	21:34:00	00 12:33:50	W	FOIA	t
194	110602	11:28:35	00 13:24:35	W	FOA	t
195	112802	17:05:49	00 04:00:00	W	UC	t
196	112802	07:49:43	00 04:00:00	W	FOIA	t
197	112802	15:36:04	00 04:00:00	W	UC	t
198	112802	06:52:41	00 04:00:00	W	FOIA	t
199	112802	11:54:45	00 04:32:04	W	FOIA	t
200	112802	08:39:39	01 20:14:54	W	UC	t
201	113902	23:32:27	00 04:00:00	W	UC	t
202	113902	16:55:50	00 04:00:00	W	FOIA	d
203	113902	05:17:05	00 04:00:00	N2	FOIA	t
204	113902	13:46:12	00 04:00:00	W	FOIA	t
205	113902	22:40:46	00 08:24:33	N2	UC	t
206	113902	16:53:42	00 06:23:24	W	FOIA	t
207	114702	20:52:30	00 04:00:00	W	FOIA	t
208	114702	14:45:03	00 04:00:00	W	FOIA	t
209	114702	04:09:15	00 04:00:00	W	UC	t
210	114702	09:50:10	00 04:00:00	W	FOIA	t
211	114702	14:27:45	00 04:00:00	W	FOIA	d
212	114702	11:03:08	00 08:11:30	W	FOIA	t
213	114702	13:27:36	00 09:30:51	W	FOIA	d
214	114702	21:04:57	00 07:07:20	W	FOIA	t
215	114902	08:30:29	00 04:00:00	W	FOA	s
216	114902	14:42:32	00 04:00:00	W	FOIA	b
217	114902	19:42:40	00 04:00:00	W	FOIA	s
218	114902	05:59:33	00 04:00:00	N2	FBTC	t
219	114902	17:18:54	00 07:19:37	W	UC	r
220	114902	11:52:26	00 18:03:32	W	FOIA	a
221	114902	09:27:30	00 15:35:47	W	FOIA	t
222	123902	02:52:47	00 04:00:00	N2	FBTC	t
223	123902	01:38:19	00 04:00:00	N2	FBTC	t
224	123902	02:11:22	00 04:00:00	R	FOIA	t
225	123902	18:57:10	00 11:32:53	W	FOIA	t
226	123902	15:22:45	00 18:39:43	W	FOA	t

S: seizure. ID: patient identifier. Seizure vigilance state: wakefulness (W), NREM sleep stage I (N1), NREM sleep stage II (N2), REM sleep stage (R). Seizure ILAE classification: focal onset aware (FOA), focal onset impaired awareness (FOIA), focal to bilateral tonic-clonic (FBTC), unclassified (UC). Seizure activity pattern: rhythmic alpha waves (a), rhythmic beta waves (b), cessation of interictal activity (c), rhythmic delta waves (d), amplitude depression (m), repetitive spiking (r), rhythmic sharp waves (s), rhythmic theta waves (t), unclear (?). Training seizures are highlighted in grey.

2 Grid search results

Table S3 and Table S4 contain average geometric mean between sample sensitivity and sample specificity obtained while searching for the best hyperparameters for the deep neural network and for the shallow artificial neural network, respectively. The selected hyperparameters are in bold.

2.1 Grid search results for deep neural network

Table S3: Results of the grid search of the optimal hyperparameters for the deep classifier.

Number of Filters (First layer)	Filter Size	LSTM Units	Avg G-Mean
32	3	32	0.291
		64	0.292
		128	0.278
	5	32	0.292
		64	0.312
		128	0.278
	7	32	0.299
		64	0.291
		128	0.275
64	3	32	0.349
		64	0.306
		128	0.300
	5	32	0.318
		64	0.315
		128	0.336
	7	32	0.295
		64	0.308
		128	0.292
**128**	**3**	32	0.354
		**64**	**0.378**
		128	0.362
	5	32	0.349
		64	0.346
		128	0.353
	7	32	0.346
		64	0.320
		128	0.316

2.2 Grid search results for shallow artificial neural network

Table S4: Results of the grid search of the optimal hyperparameters for the shallow classifier.

Number of neurons in fully connected layer (FC layer)	Geometric- Mean
**None**	**0.331**
8	0.326
16	0.320
32	0.312
64	0.311
128	0.301
256	0.307

3 Results obtained for all approaches

Table S5 and Table S6 contain seizure sensitivities and false prediction rate per hour (FPR/h) for the approaches developed using denoised EEG data and noisy EEG data, respectively. Table S7 contains results obtained by Pinto *et al.*[3,4].

3.1 Learning curves of the chronological approach

Fig. S1 presents the learning curves obtained for one deep learning model applied to one patient using the chronological approach. As seen in the figure, the validation loss curves improve as the training sets become larger, indicating that the model is able to achieve better generalisation.

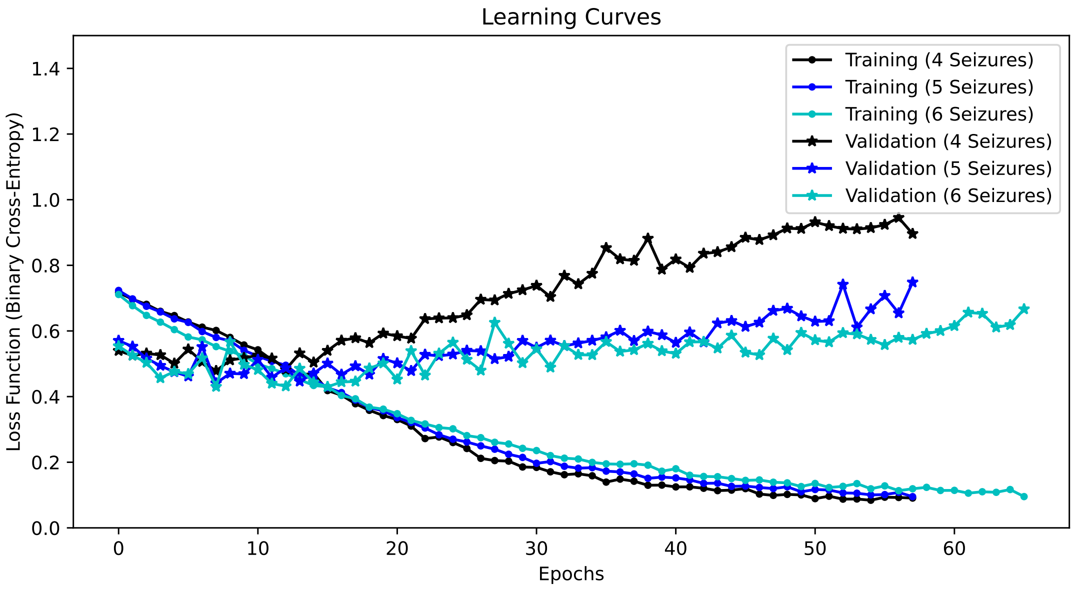

Fig. S1. Example of learning curves obtained for one model of one patient with 3 test seizures considering the chronological approach. The black lines represent the training and validation curves using 4 training seizures.The black lines represent the training and validation curves using 5 training seizures. The cyan lines represent the training and validation curves using 6 training seizures.

3.2 Results obtained for approaches using denoised data

Table S5: Results for each patient using denoised data.

	Denoised $EEG_{Standard}$			Denoised $EEG_{Chronological}$			Denoised $Features_{Standard}$			Denoised $Features_{Chronological}$		
**Patient**	**Sensitivity**	**FPR/h**	**Above Chance Level**	**Sensitivity**	**FPR/h**	**Above Chance Level**	**Sensitivity**	**FPR/h**	**Above Chance Level**	**Sensitivity**	**FPR/h**	**Above Chance Level**
**402**	0.00	1.316	0	0.00	0.331	0	0.00	1.474	0	0.00	0.541	0
**8902**	0.50	0.105	1	0.50	0.161	1	1.00	0.163	1	0.50	0.220	1
**11002**	0.50	0.062	1	0.50	0.130	1	0.50	0.370	1	0.50	0.370	1
**16202**	0.33	0.401	1	0.33	0.496	1	0.00	2.484	0	0.00	2.486	0
**21902**	0.50	0.068	1	0.50	0.068	1	0.50	0.880	1	0.50	1.230	1
**23902**	0.00	0.482	0	0.00	0.483	0	0.50	1.321	1	0.50	1.332	0
**26102**	0.50	1.003	1	0.50	0.828	1	0.50	0.882	1	0.50	0.988	1
**30802**	0.33	0.137	1	0.33	0.138	1	0.67	0.373	1	0.67	0.373	1
**32702**	0.00	0.000	0	0.00	0.123	0	0.00	0.192	0	0.00	0.192	0
**45402**	0.50	1.598	0	0.50	0.503	1	1.00	3.176	1	1.00	2.194	1
**46702**	0.00	1.145	0	0.00	0.342	0	0.00	3.289	0	0.00	2.588	0
**50802**	0.00	0.134	0	0.50	0.657	1	0.00	0.134	0	0.00	0.211	0
**52302**	0.00	0.000	0	0.00	0.000	0	0.00	0.000	0	0.00	0.000	0
**53402**	0.00	0.290	0	0.00	0.232	0	0.00	0.384	0	0.00	0.415	0
**55202**	0.33	0.202	1	0.33	0.344	1	0.33	1.277	0	0.00	1.701	0
**56402**	0.00	0.055	0	0.00	0.213	0	0.50	2.812	0	0.50	3.411	0
**58602**	0.00	0.121	0	0.00	0.121	0	0.00	0.567	0	0.00	0.567	0
**59102**	1.00	2.227	1	0.50	0.207	1	1.00	3.048	1	1.00	0.997	1
**60002**	0.00	0.151	0	0.00	0.124	0	0.00	2.556	0	0.50	1.614	1
**64702**	0.00	0.000	0	0.00	0.000	0	0.00	0.334	0	0.00	0.334	0
**75202**	0.00	0.417	0	0.00	0.305	0	0.00	0.207	0	0.00	0.304	0
**80702**	0.00	0.059	0	0.00	0.124	0	1.00	2.109	1	1.00	1.846	1
**81102**	0.00	0.207	0	0.00	0.207	0	1.00	0.648	1	1.00	0.648	1
**85202**	0.00	0.000	0	0.00	0.055	0	0.50	0.312	1	1.00	0.316	1
**93402**	0.00	0.179	0	0.50	0.179	1	0.50	0.256	1	1.00	0.156	1
**93902**	0.50	0.190	1	0.50	0.734	1	0.50	0.576	1	0.50	0.945	1
**94402**	0.00	0.372	0	0.00	0.372	0	0.00	1.024	0	0.00	1.069	0
**95202**	0.33	0.387	1	0.33	0.549	1	0.67	0.524	1	0.67	0.604	1
**96002**	0.00	0.051	0	0.33	0.204	1	0.67	0.586	1	0.67	0.651	1
**98102**	0.00	0.000	0	0.00	0.000	0	0.50	0.825	1	0.50	0.822	1
**98202**	0.00	0.159	0	0.00	0.159	0	0.00	0.732	0	0.00	0.732	0
**101702**	0.00	0.000	0	0.00	0.000	0	0.50	0.212	1	0.50	0.154	1
**102202**	0.33	0.311	1	0.33	0.205	1	0.00	1.071	0	0.00	0.996	0
**104602**	0.00	0.000	0	0.00	0.000	0	0.00	0.079	0	0.00	0.499	0
**109502**	0.00	0.000	0	0.00	0.213	0	0.00	0.000	0	0.50	1.060	1
**110602**	0.00	0.000	0	0.00	0.000	0	0.50	0.570	1	0.00	0.431	0
**112802**	0.50	0.434	1	0.50	0.729	1	0.00	0.868	0	0.50	1.068	1
**113902**	0.00	0.271	0	0.00	0.172	0	0.50	0.083	1	0.50	0.083	1
**114702**	0.00	0.047	1	0.33	0.000	1	0.33	0.337	1	0.33	0.746	1
**114902**	0.00	0.000	0	0.00	0.000	0	0.33	0.084	1	0.33	0.241	1
**123902**	0.00	0.000	0	0.00	0.000	0	0.00	0.000	0	0.00	0.000	0
**Total**	**0.150±0.244**	**0.307±0.482**	**12 (0.29)**	**0.179±0.222**	**0.237±0.225**	**17 (0.41)**	**0.341±0.350**	**0.898±0.955**	**21 (0.51)**	**0.370±0.360**	**0.857±0.770**	**22 (0.54)**

3.3 Results obtained for approaches using noisy data

Table S6: Results for each patient using noisy data.

	Noisy EEG$_{Standard}$			Noisy EEG$_{Chronological}$			Noisy Features$_{Standard}$			Noisy Features$_{Chronological}$		
**Patient**	**Sensitivity**	**FPR/h**	**Above Chance Level**	**Sensitivity**	**FPR/h**	**Above Chance Level**	**Sensitivity**	**FPR/h**	**Above Chance Level**	**Sensitivity**	**FPR/h**	**Above Chance Level**
**402**	0.00	1.655	0	0.00	0.728	0	0.00	1.461	0	0.00	0.709	0
**8902**	0.50	0.105	1	0.50	0.163	1	1.00	0.164	1	1.00	0.164	1
**11002**	0.00	0.130	0	0.50	0.130	1	0.00	0.358	0	0.00	0.272	0
**16202**	0.00	0.311	0	0.00	0.405	0	0.00	1.455	0	0.00	1.553	0
**21902**	0.00	0.403	0	0.00	0.403	0	0.50	0.866	1	0.50	1.209	1
**23902**	0.00	0.280	0	0.00	0.381	0	0.00	1.698	0	0.00	1.453	0
**26102**	0.50	0.939	1	0.50	1.000	1	0.50	0.823	1	0.50	1.200	1
**30802**	0.33	0.137	1	0.33	0.108	1	0.67	0.335	1	0.67	0.299	1
**32702**	0.00	0.059	0	0.00	0.123	0	0.00	0.192	0	0.00	0.192	0
**45402**	0.50	2.459	0	0.50	0.508	1	1.00	2.889	1	1.00	2.037	1
**46702**	0.00	1.453	0	0.00	0.842	0	0.00	2.725	0	0.00	2.241	0
**50802**	0.00	0.064	0	0.50	0.484	1	0.00	0.134	0	0.00	0.171	0
**52302**	0.00	0.000	0	0.00	0.000	0	0.00	0.000	0	0.00	0.000	0
**53402**	0.00	0.229	0	0.00	0.202	0	0.00	0.316	0	0.00	0.319	0
**55202**	0.33	0.202	1	0.33	0.141	1	1.00	0.976	1	0.67	1.253	1
**56402**	0.00	0.085	0	0.00	0.249	0	0.00	1.954	0	0.00	2.188	0
**58602**	0.00	0.121	0	0.00	0.121	0	0.50	0.261	1	0.50	0.619	1
**59102**	1.00	2.137	1	0.50	0.224	1	1.00	2.719	1	0.50	1.088	0
**60002**	0.00	0.235	0	0.50	0.151	1	0.50	1.940	0	0.50	1.081	1
**64702**	0.00	0.000	0	0.00	0.000	0	0.00	0.195	0	0.00	0.195	0
**75202**	0.00	0.307	0	0.00	0.304	0	0.33	5.549	0	0.00	1.633	0
**80702**	0.00	0.619	0	0.00	0.422	0	1.00	2.129	1	1.00	1.824	1
**81102**	0.00	0.268	0	0.00	0.268	0	1.00	0.643	1	1.00	0.643	1
**85202**	0.00	0.000	0	0.00	0.000	0	0.50	0.315	1	0.50	0.311	1
**93402**	0.00	0.132	0	0.50	0.156	1	1.00	0.540	1	1.00	0.439	1
**93902**	0.00	0.183	0	0.50	0.916	1	0.50	0.578	1	0.50	0.757	1
**94402**	0.00	0.298	0	0.00	0.452	0	0.00	0.709	0	0.00	1.209	0
**95202**	0.67	0.579	1	0.67	0.526	1	0.67	0.524	1	0.67	0.604	1
**96002**	0.00	0.000	0	0.00	0.000	0	0.67	0.583	1	0.67	0.782	1
**98102**	0.00	0.075	0	0.00	0.024	0	0.50	0.670	1	0.50	0.816	1
**98202**	0.00	0.000	0	0.00	0.000	0	0.00	0.116	0	0.00	0.116	0
**101702**	0.00	0.000	0	0.00	0.000	0	0.00	0.099	0	0.00	0.154	0
**102202**	0.33	0.239	1	0.00	0.141	0	0.00	1.070	0	0.33	1.059	1
**104602**	0.00	0.000	0	0.00	0.169	0	0.00	0.000	0	0.00	0.382	0
**109502**	0.00	0.000	0	0.00	0.154	0	0.00	0.000	0	0.50	0.997	1
**110602**	0.00	0.000	0	0.00	0.000	0	0.50	0.570	1	0.00	0.365	0
**112802**	0.50	0.509	0	0.00	0.148	0	0.00	1.242	0	0.00	1.984	0
**113902**	0.50	0.082	1	0.50	0.082	1	0.50	0.083	1	0.50	0.083	1
**114702**	0.00	0.047	0	0.33	0.151	1	0.67	0.828	1	0.33	1.177	1
**114902**	0.00	0.000	0	0.00	0.000	0	0.33	0.212	1	0.33	0.396	1
**123902**	0.00	0.000	0	0.00	0.000	0	0.00	0.000	0	0.00	0.000	0
**Total**	**0.126±0.244**	**0.350±0.575**	**8 (0.20)**	**0.163±0.234**	**0.251±0.261**	**14 (0.34)**	**0.362±0.384**	**0.925±1.090**	**20 (0.49)**	**0.333±0.356**	**0.829±0.647**	**21 (0.51)**

3.4 Results obtained in other studies using EEG data from patients from EPILEPSIAE database

Table S7: Results for each patient obtained in studies of Pinto *et al.*[3,4].

	Pinto *et al.* (2021)[3]			Pinto *et al.* (2022)[4]		
**Patient**	**Sensitivity**	**FPR/h**	**Above Chance Level**	**Sensitivity**	**FPR/h**	**Above Chance Level**
**402**	-	-	-	-	-	-
**8902**	-	-	-	0.20	0.16	0
**11002**	0.27	1.57	0	0.00	0.27	0
**16202**	0.30	0.49	0	-	-	-
**21902**	-	-	-	0.00	0.18	0
**23902**	-	-	-	0.08	0.22	0
**26102**	-	-	-	0.13	0.34	0
**30802**	0.58	3.77	1	0.19	0.19	0
**32702**	-	-	-	-	-	-
**45402**	-	-	-	-	-	-
**46702**	-	-	-	0.25	0.28	1
**50802**	-	-	-	0.03	0.22	0
**52302**	-	-	-	0.01	0.24	0
**53402**	0.63	0.50	1	0.30	0.26	1
**55202**	0.70	1.21	1	0.18	0.23	0
**56402**	-	-	-	0.05	0.14	0
**58602**	0.38	2.14	0	0.05	0.25	0
**59102**	-	-	-	-	-	-
**60002**	0.48	1.29	0	-	-	-
**64702**	0.02	0.92	0	0.08	0.14	0
**75202**	0.70	0.69	1	0.19	0.14	0
**80702**	0.31	0.65	0	0.10	0.14	0
**81102**	-	-	-	-	-	-
**85202**	0.47	0.36	1	0.42	0.25	1
**93402**	-	-	-	0.11	0.32	0
**93902**	-	-	-	0.37	0.23	1
**94402**	0.38	1.05	0	0.13	0.36	0
**95202**	0.14	0.66	0	0.09	0.16	0
**96002**	0.23	0.77	0	0.16	0.22	0
**98102**	-	-	-	0.32	0.11	1
**98202**	0.19	1.72	0	-	-	-
**101702**	0.23	0.44	0	0.34	0.24	1
**102202**	-	-	-	0.22	0.18	1
**104602**	-	-	-	0.33	0.26	1
**109502**	0.52	1.29	0	0.11	0.14	0
**110602**	0.47	0.33	1	0.37	0.20	1
**112802**	-	-	-	-	-	-
**113902**	-	-	-	0.28	0.07	1
**114702**	-	-	-	0.16	0.35	1
**114902**	0.31	0.25	1	0.33	0.10	1
**123902**	-	-	-	0.07	0.14	0
**Total**	**0.38±0.19**	**1.06±0.84**	**8 (0.37)**	**0.18±0.12**	**0.21±0.07**	**12 (0.38)**

3.5 Results obtained using other machine learning models

To ensure the robustness of our study, we evaluated the effectiveness of denoised data and chronological training on other previously proposed seizure prediction models. Specifically, we selected a deep learning model proposed by Truong *et al.*[5] and a shallow classifier model with handcrafted features proposed by Karoly*et al.*[6]. The deep learning model comprises a convolutional neural network (CNN) that takes spectrograms obtained from EEG windows using short-term Fourier transform. In the case of the shallow model, some features were firstly extracted by the 19 channels, such as the energy from several frequency bands (8-16 Hz, 16-32 Hz, 32-64 Hz, 64-90 Hz) and the line length. The Kullback-Leibler distance method is then used to select the best features, which are subsequently used to develop the logistic regression.

Table S8: Average results of the additional seizure prediction models, for all 41 patients. The models that used EEG time series were developed using a convolutional neural network with spectrograms as input as performed in Truong *et al.*[5]. The models that used features were developed using a logistic regression with the same features extracted by Karoly *et al.*[6].

Approach	Seizure Sensitivity	FPR/h	Above Chance Level (%)
**Denoised EEG$_{Standard}$ - CNN (Spectrograms)**	0.13±0.27	0.18±0.38	9 (0.22)
**Denoised EEG$_{Chronological}$ - CNN (Spectrograms)**	0.14±0.27	0.11±0.16	11 (0.27)
**Denoised Features$_{Standard}$ - Logistic regression (Features)**	0.44±0.37	1.91±0.96	19 (0.46)
**Denoised Features$_{Chronological}$ - Logistic regression (Features)**	0.42±0.36	1.97±2.51	19 (0.54)
**Noisy EEG$_{Standard}$ - CNN (Spectrograms)**	0.10±0.22	0.18±0.46	8 (0.20)
**Noisy EEG$_{Chronological}$ - CNN (Spectrograms)**	0.11±0.23	0.09±0.15	9 (0.22)
**Noisy Features$_{Standard}$ - Logistic regression (Features)**	0.44±0.40	2.58±3.27	16 (0.39)
**Noisy Features$_{Chronological}$ - Logistic regression (Features)**	0.38±0.34	2.32±2.84	13 (0.32)

References

1. R. S. Fisher, J. H. Cross, J. A. French, N. Higurashi, E. Hirsch, F. E. Jansen, L. Lagae, S. L. Moshé, J. Peltola, E. Roulet Perez, I. E. Scheffer, and S. M. Zuberi, "Operational classification of seizure types by the International League Against Epilepsy: Position Paper of the ILAE Commission for Classification and Terminology," *Epilepsia*, vol. 58, pp. 522–530, 4 2017.

2. P. J. Karoly, V. R. Rao, N. M. Gregg, G. A. Worrell, C. Bernard, M. J. Cook, and M. O. Baud, "Cycles in epilepsy," *Nature Reviews Neurology*, vol. 17, pp. 267–284, 5 2021.

3. M. F. Pinto, A. Leal, F. Lopes, A. Dourado, P. Martins, and C. A. Teixeira, "A personalized and evolutionary algorithm for interpretable EEG epilepsy seizure prediction," *Scientific Reports*, vol. 11, p. 3415, 12 2021.

4. M. Pinto, T. Coelho, A. Leal, F. Lopes, A. Dourado, P. Martins, and C. Teixeira, "Interpretable EEG seizure prediction using a multiobjective evolutionary algorithm," *Scientific Reports*, vol. 12, p. 4420, 12 2022.

5. N. D. Truong, A. D. Nguyen, L. Kuhlmann, M. R. Bonyadi, J. Yang, S. Ippolito, and O. Kavehei, "Convolutional neural networks for seizure prediction using intracranial and scalp electroencephalogram," *Neural Networks*, vol. 105, pp. 104–111, 2018.

6. P. J. Karoly, H. Ung, D. B. Grayden, L. Kuhlmann, K. Leyde, M. J. Cook, and D. R. Freestone, "The circadian profile of epilepsy improves seizure forecasting," *Brain*, vol. 140, no. 8, pp. 2169–2182, 2017.
